# Proteomic Characterization of *Drosophila melanogaster* Proboscis

**DOI:** 10.3390/biology11111687

**Published:** 2022-11-21

**Authors:** Enisa Aruçi, Jean-Michel Saliou, Jean-François Ferveur, Loïc Briand

**Affiliations:** 1 Centre des Sciences du Goût et de l’Alimentation, CNRS, INRAE, Institut Agro, Université Bourgogne Franche-Comté, 21000 Dijon, France; 2 CNRS, INSERM CHU Lille, Institut Pasteur de Lille, UAR CNRS 2014–US Inserm 41–PLBS, University of Lille, 59000 Lille, France

**Keywords:** chemosensory system, taste, odorant-binding proteins, mass spectrometry, proteomics

## Abstract

**Simple Summary:**

Insects use chemical signals to locate food, interact with their environment, and for social communication. More precisely, the peripheral sensory olfactory and gustatory systems allow the detection of chemical signals coming from the environment. Volatile or sapid molecules enter the sensory organs and are transported by specialized proteins through the internal aqueous phase to the chemosensory receptors. We used proteomic analysis to identify the soluble proteins in the proboscis, an organ that is part of the gustatory system of *Drosophila melanogaster.* A total of 586 proteins were identified, and 19 proteins were used for further analysis. We identified two proteins implicated in the transport of molecules, such as the odorant-binding proteins OBP19d and OBP56d. Interestingly, OBP56d showed higher expression in female Drosophila, whereas OBP19d showed no sex differences. We also identified proteins implicated in the metabolism of chemicals. Other molecules, such as pheromones, were detected by the proboscis, but our analysis did not identify any of them. In conclusion, we found that the proboscis was involved in the detection of many classes of molecules that can impact feeding and sexual behavior in *Drosophila melanogaster*.

**Abstract:**

*Drosophila melanogaster* flies use their proboscis to taste and distinguish edible compounds from toxic compounds. With their proboscis, flies can detect sex pheromones at a close distance or by contact. Most of the known proteins associated with probosci’s detection belong to gustatory receptor families. To extend our knowledge of the proboscis-taste proteins involved in chemo-detection, we used a proteomic approach to identify soluble proteins from Drosophila females and males. This investigation, performed with hundreds of dissected proboscises, was initiated by the chromatographic separation of tryptic peptides, followed by tandem mass spectrometry, allowing for femtomole detection sensitivity. We found 586 proteins, including enzymes, that are involved in intermediary metabolism and proteins dedicated to various functions, such as nucleic acid metabolism, ion transport, immunity, digestion, and organ development. Among 60 proteins potentially involved in chemosensory detection, we identified two odorant-binding proteins (OBPs), i.e., OBP56d (which showed much higher expression in females than in males) and OBP19d. Because OBP56d was also reported to be more highly expressed in the antennae of females, this protein can be involved in the detection of both volatile and contact male pheromone(s). Our proteomic study paves the way to better understand the complex role of Drosophila proboscis in the chemical detection of food and pheromonal compounds.

## 1. Introduction

**Figure 1:**
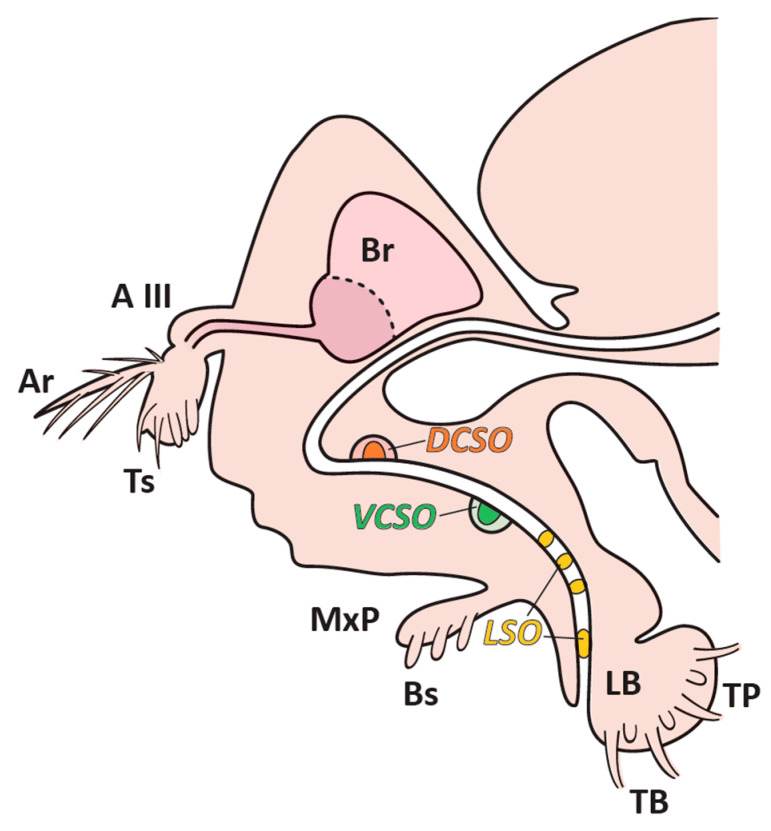
*Drosophila melanogaster* head with olfactory organs, antennae, maxillary palps (Mxp), gustatory organ, and propobscis presented with their sensilla. Antennae with third antennal segment (A III), arista (Ar), and trichoid sensillum (Ts). Mxp with basiconic sensillum (Bs). The labellum (LB) with taste bristle (TB) and taste peg (TP), and three internal taste organs indicated (i) the labral sense organs (LSOs) in yellow; (ii) the ventral cibarial sense organ (VSCO) in green; and (iii) the dorsal cibarial sense organ (DSCO) in orange. Brain (Br) (Figure adapted from [2]).

Insects use their taste system to evaluate food quality and discriminate between edible nutrients and toxic ones. The taste system of the model insect *Drosophila melanogaster* is distributed on multiple parts and appendages of the fly body (proboscis, legs, wing margins, and ovipositor [1,2,3]). The proboscis, a foldable appendage extending from the adult head, includes external and internal taste organs. The external taste organs consist of two labella covered with taste sensory hairs, while the internal taste organs consist of three groups of pharyngeal sensory cells: the labral sense organs (LSOs), the dorsal cibarial sense organ (DCSO), and the ventral cibarial sense organ (VCSO) (Figure 1). Sapid molecules are initially detected by the sensory neurons housed in the sensilla covering the proboscis, and their quality is then evaluated by pharyngeal organs. Each taste sensillum is a uni-porous sensillum containing two or four chemosensory neurons whose dendrites harbor highly specialized receptors, such as gustatory receptors (GRs; [4]), ionotropic receptors (IRs; [5]), members of the transient receptor potential family (TRP; [6]), takeout-like receptors [7], and Pickpocket receptors (ppk; [8]). All sensilla also house one mechanosensory neuron. The electrophysiological recording of gustatory sensilla stimulated with a variety of taste stimuli revealed the presence of four types of gustatory receptor neurons (GRNs) that are either sensitive to (1) sugar, (2) water, (3) low or (4) high salt concentrations [9,10,11]. The dendrites of all these neurons are bathed in hydrophilic sensillar lymph. This sensillar lymph contains three large families of proteins: xenobiotic metabolizing enzymes (XMEs; [12,13], and odorant-binding proteins (OBPs; [13,14,15]), all of which have been hypothesized to be involved in the transport and/or elimination of chemical stimuli and chemosensory proteins (CSPs; [16]). CSPs show a broad tissue distribution because they are expressed both in the sensory and non-sensory organs involved in various functions during development [14,17,18]. However, there is no clear evidence that they participate in olfaction or in gustation. Nevertheless, in Drosophila, 4 CSPs were identified, which might be involved in the storage and release of pheromone molecules [19]. XMEs are involved in detoxification processes and metabolite elimination [12,20]. OBPs are small soluble proteins that are found in high concentrations in the sensillar lymph of insects [19]. Fifty-two OBP-coding genes have been identified in *Drosophila melanogaster*. Several OBPs were found in the non-sensory tissues, such as the Drosophila male reproductive tract [21,22], hindgut [23], and fat body [24]. OBPs can be also expressed during preimaginal development in Drosophila larvae [18].

The best-documented function of OBPs is their role in the transport of lipophilic ligands across the aqueous sensilla lymph fluid to the dedicated sensory receptors [15,17], [25,26]. Several OBPs were shown to play nonolfactory roles [18]. Among them, OBP19c, OBP56g, OBP56b, OBP57b, OBP57e, and OBP83c were found to be only expressed in the gustatory organs [27]. Moreover, OBP49a, which is expressed in the accessory cells surrounding sweet-responding GRNs, is required to suppress the attractive sugar response after exposure to bitter compounds [28]. Both leg-expressed OBP57d and OBP57e respond in a species-specific manner to C6-C9 fatty acids and elicit egg-laying behavior [29]. Additionally, the proboscis-expressed OBP19b is necessary to transport essential amino acids [30]. OBP59a, which is expressed in the second antennal segment, is implicated in hygrosensation [31,32]. Other intracellular functions of OBPs remain elusive [33].

To obtain a broader picture of the soluble proteins involved in the chemoreception expressed in the *Drosophila melanogaster* proboscis and to identify OBPs and XMEs present in this major taste appendage, we used a proteomics approach. This technique is based on the separation of tryptic peptides by nano-LC, followed by tandem mass spectrometry (MS/MS) with femtomole detection sensitivities. We compared the pattern of identified proteins between the female and male flies. We found several proteins that were potentially involved in taste, some of which showed sex-specific expression. These proteins include two OBPs, one of which showed clear sexual dimorphism.

## 2. Materials and Methods

### 2.1. Drosophila Stocks and Sample Preparation

The Canton-S *Drosophila melanogaster* strain (Cs) was kept in a room at 24 ± 0.5 °C and 65 ± 5% relative humidity with a 12 h light/dark cycle. Flies were raised on standard medium culture (1.5% agar-agar, 10% brewer’s yeast, 9% corn flour, 0.4% methyl para-hydroxy-benzoate) to ensure development. Virgin female or male flies aged 4 days were dissected. The proboscis was hand-dissected under a microscope and placed in an Eppendorf tube containing 250 µL of 10 mM Tris at pH 8. We obtained duplicate pools of 350 or 550 proboscides dissected from the virgin females or virgin males, respectively. Of note, the pair of maxillary palps, normally present on the proboscis, was discarded. These samples were kept on ice and submitted to an ultrasound treatment for 2 min with 5 s pulses every 3 s at 30 W (2 times). The soluble fraction was separated from the cell debris, or non-soluble material, by centrifugation at 20,000× *g* for 30 min at 4 °C. To concentrate all the proteins, we performed polyacrylamide gel electrophoresis (SDS-PAGE). A 12% separation gel was used (40% polyacrylamide; Tris 1.5 M pH 8.8; H_2_O; 10% SDS; N,N,N,N-tetramethylethyl ethyllenediamine (Temed) and ammonium persulfate (Aps) (10%), and a 12% concentration gel was used (40% polyacrylamide; Tris 0.5 M pH 8.8; H_2_O; 10% SDS; Temed and Aps 10%). Twenty microliters of this sample (15 µL of soluble fraction and 5 µL of TDD) were deposited on a gel, and migration was performed at 200 V and 20 mA. Once the samples migrated to the concentration gel, the migration was stopped, and we cut out the band samples in the gel and tested them by mass spectrometry proteomic analysis.

### 2.2. Mass Spectrometry Proteomic Analysis

The proteins were loaded on SDS-PAGE to perform one gel slice trypsin digestion for each sample. The peptides were extracted with 0.1% diluted in formic acid in acetonitrile and evaporated to a volume of 8 µL, which was finally injected into an UltiMate 3000 RSLCnano System (Thermo Fisher Scientific, Waltham, MA, USA). The peptides were automatically fractionated onto a commercial C18 reversed-phase column (75 μm × 500 mm, 2-μm particle, PepMap100 RSLC column, Thermo Fisher Scientific, temperature 55 °C). Trapping was performed for 4 min at 5 μL/min with solvent A (98% H_2_O, 2% acetonitrile, and 0.1% formic acid). The peptides were eluted using two solvents, A (0.1% formic acid in water) and B (0.1% formic acid in acetonitrile), at a flow rate of 300 nL/min. Gradient separation lasted 3 min in 3% B, 170 min from 3% to 20% B, and 20 min from 20% to 80% B, and was maintained for 15 min at 80% B. The column was equilibrated for 17 min with 3% buffer B prior to the next sample analysis. The eluted peptides from the C18 column were analyzed by Q-Exactive instruments (Thermo Fisher Scientific). The electrospray voltage was 1.9 kV, and the capillary temperature was 275 °C. Full MS scans were acquired in the Orbitrap mass analyzer over the *m*/*z* 400–1200 range with a 70,000 (*m*/*z* 200) resolution. The target value was 3.00E^06^. The fifteen most intense peaks with charge states between 2 and 5 were fragmented in the higher-energy collision-activated dissociation cell with a normalized collision energy of 27%, and the tandem mass spectrum was acquired in the Orbitrap mass analyzer with a 17 500 (*m*/*z* 200) resolution. The target value was 1.00E^05^. The ion selection threshold was 5.0E^04^ counts, and the maximum allowed ion accumulation time was 250 ms for full MS scans and 100 ms for tandem mass spectra. The dynamic exclusion was set to 30 s.

### 2.3. Proteomic Data Analysis

Raw data collected during nano LC–MS/MS analyses were processed and converted into an *.mgf peak list format with Proteome Discoverer 1.4 (Thermo Fisher Scientific). MS/MS data were analyzed using the search engine Mascot (version 2.4.0, Matrix Science, London, UK) installed on a local server. Searches were performed with a tolerance on the mass measurement of 10 ppm for the precursor and 0.02 Da for fragment ions against a composite target-decoy database (6064 × 2 total entries) built with a Drosophila Swissprot database (taxonomy 7215, December 2020, 5946 entries) and fused with the sequences of recombinant trypsin and a list of classical contaminants (118 entries). Cysteine carbamidomethylation, methionine oxidation, protein N-terminal acetylation, and cysteine propionamidation were searched as variable modifications. Up to one missed trypsin cleavage was allowed. The identification results were imported into Proline software installed on a local server (http://proline.profiproteomics.fr accessed on 12 October 2022) [34] for validation. Peptide spectrum matches (PSM) taller than 9 residues and ion scores >15 were retained. The false discovery rate was then optimized to be below 1% at the protein level using the Mascot Modified Mudpit score. Peptide abundances were extracted with Proline software version 2.0 using an *m*/*z* tolerance of 10 ppm. The alignment of the LC-MS runs were performed using Loess smoothing. Cross-assignment of the peptide ion abundances was performed among the samples and controls using an *m*/*z* tolerance of 10 ppm and a retention time tolerance of 60 s. Protein abundances were computed using the median ratio fitting of the unique peptide abundances normalized at the peptide level using the median.

## 3. Results

### 3.1. Broad Identification of Soluble Proteins from Drosophila melanogaster Proboscis

**Figure 2:**
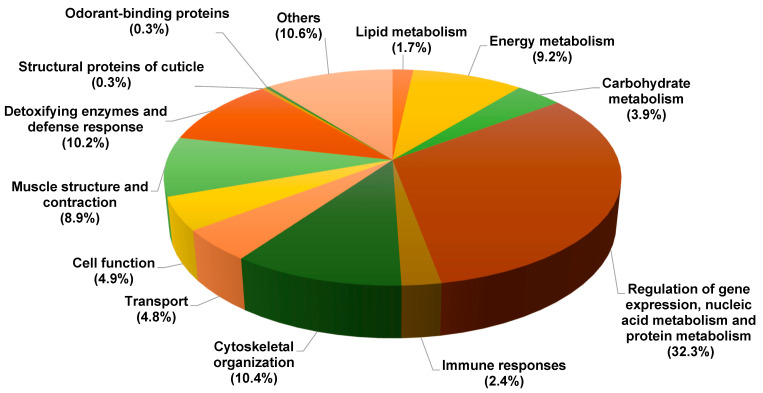
**Distribution of all proteins identified in *Drosophila melanogaster* proboscis (proteins identified in females and males are pooled here)**. The 586 proteins belong to 13 functional categories shown in this pie chart representation (the percentage indicated for each category is relative to the total number of proteins; each protein was counted in only one category).

Four proboscis samples (two for each sex) were hand-dissected in four-day-old flies (yielding four pools of approximately 350 females or 550 males). Based on their presence in at least one of the four pools of dissected proboscis, we identified a total of 586 soluble proteins (Table 1 and Table S1). The analysis of the identified proteins suggested that they originated either from the cytoplasm of disrupted cells or from the extracellular sensillar lymph of taste sensilla. Here, we only show the proteins detected in both samples for each sex. These proteins were classified into 13 categories according to their main known functions (Figure 2). Among all the identified proteins, 32.3% are involved in gene expression regulation, nucleic acid metabolism, and protein metabolism. These proteins are involved in the regulation of transcription, histone modification, mRNA splicing, and protein degradation. We identified actin-, tubulin-, and myosin-binding proteins that were involved in the formation of the cytoskeleton structure. They were classified in the “Cytoskeleton organization” category (10.4%). We found that proteins involved in cell differentiation or in the regulation of the membrane trafficking pathway from the Golgi apparatus were classified in the “Cell function” category (4.8%). We identified proteins involved in electron or proton transportation which played a major role in muscle contraction together with the proteins present in the actin and thick myosin filaments, which were classified in the” Muscle structure and contraction” category (8.9%).

We also found that 1.7%, 9.2%, and 3.9% of the proteins were involved in the metabolism of lipids, energy, and carbohydrates, respectively. All the above-mentioned proteins are implicated in fatty acid cycles, mitochondrial activity, or the glucose cycle. Only 0.3% of the identified proteins were involved in transportation, i.e., the category of “Odorant-binding proteins”. We identified the protective enzymes associated with antibacterial defense and response against oxidative damage. They were classified into the “Detoxifying enzymes and defense” category (10.4%). We also found two groups of proteins potentially related to chemoreception: (1) proteins involved in the transport of volatile or sapid molecules and (2) enzymes involved in detoxification processes called XMEs. Other proteins, either with another function or with a yet unknown function, were classified in the “Others” category (10.6%).

### 3.2. Identification of Proteins Involved in Transport and Detoxification Metabolism

**Figure 3:**
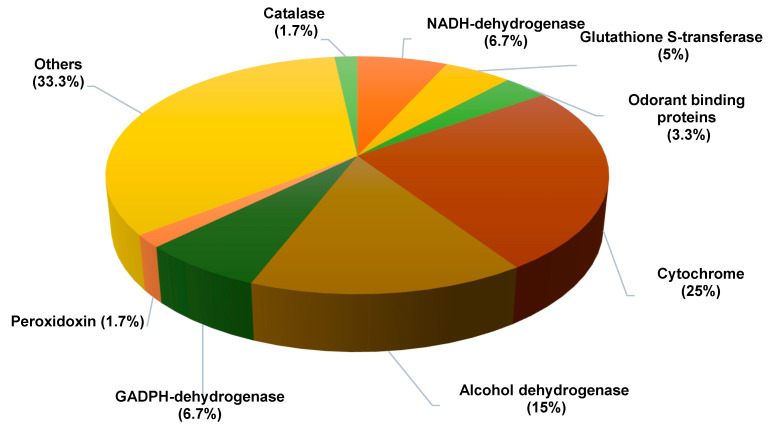
**Functional distribution of the 60 proteins putatively involved in “chemoreception”**. The 60 proteins identified were classified into eight categories (the percentage indicated for each category is relative to the total number of proteins; each protein was counted in only one category).

Based on the analysis of the four samples (two female biological replicates and two male biological replicates), we identified 60 proteins potentially involved in chemoreception (Figure 3). These proteins are thought to act in the transport of chemicals and/or to metabolize and eliminate chemicals present in the proboscis. They are shown in Table 1.

To refine our analysis, we only kept the proteins that were simultaneously detected in the two biological replicates, at least in one sex. This procedure yielded a short list of 19 proteins that were putatively involved in chemo-detection: 17 were involved in detoxification and two in ligand transport (Table 2). Among the detoxification proteins, two were implicated in oxidative stress resistance (catalase, peroxiredoxin), while 15 others belonged to one of the three following categories: cytochrome (CYP), alcohol dehydrogenase (ADH), or glutathione S-transferase (GST).

More precisely, we found nine cytochromes: five P450 cytochromes and four cytochrome oxidases. The intersex comparison revealed that two P450s (CYP6d5 and CYP313a1) showed higher measured abundance in males than in females, whereas three cytochrome oxidases were more frequently identified in females than in males (Table 2). The remaining cytochromes showed no obvious difference in the measured abundance. Among the three identified GSTs, only GSTD1 was taken into account for other analyses. It was equally expressed in both sexes. Two OBPs were identified in the proboscis (OBP19d and OBP56d), with OBP56d showing a higher abundance in female proboscis than in male proboscis 5.09 × 10^6^ and 6.41 × 10^5^, respectively; Table 2).

## 4. Discussion

CYPs are often reported to be broadly tuned detoxifying enzymes [12]. More than eighty P450 cytochrome genes have been identified in *Drosophila melanogaster.* Sex-specific differences in cytochromes can be related to their implication in the metabolism of hormone precursors, pheromones, or lipids [35]. In Drosophila, it was shown that the loss of function in the P450 cytochromes induces specific defects in the mechano- and chemosensory perception [36]. CYP303a1 is essential for the development and structure of external sensory organs that mediate the reception of mechanosensory and chemosensory stimuli. This P450 cytochrome, which is only expressed in sensory bristles, may play a role in morphogenesis and cell differentiation [36]. ADH enzymes of classes 1, 2, and 3 were also identified in our proteomic analysis. Among the five ADH enzymes identified in both sexes, two showed higher expression in females (ADH and ADH class 3). ADHs are involved in the metabolic processing of alcohols and aldehydes. They were shown to be necessary for ethanol detection. For example, the level of ADH activity affects the Drosophila oviposition preference for food containing a relatively high ethanol concentration [37]. GSTs have ubiquitously expressed enzymes that are required in the detoxification processes. They catalyze the conjugation of glutathione into various molecules. The Drosophila GSTD1 enzyme is involved in the resistance to the insecticide dichloro-diphenyl-trichloroethane (DDT) [38]. It is classified in the Delta group of GSTs and appears to be highly expressed in antennae and proboscis [39]. The presence of GSTD1 was identified in other non-chemosensory organs, such as in the gut, carcass, and head without appendages, suggesting that it has a general function and is important for insecticide resistance [40,41].

The two OBPs identified in the proboscis (OBP19d and OBP56d) are not exclusive to the taste system because they are also expressed in the olfactory system [42,43]. Our analysis did not reveal sex differences in OBP19d expression. A similar result was found in *D. melanogaster* antennae [42]. In this species, OBP19d has localized, both in the hair-shaped and peg-type taste sensilla [44]. OBP19d is abundantly expressed in all adult gustatory organs on the labellum, legs, and wings and in the internal taste organs on the proboscis. In the hair-shaped sensilla, OBP19d is localized in the crescent-shaped lumen of the sensilla but not in the lumen where the dendrites of the gustatory neurons are found, suggesting that a function in the stimulus transport is unlikely in these sensilla [44]. This indicates that OBPs have roles other than the transport of lipophilic tastant ligands.

OBP56d showed a higher abundance in female proboscis than in male proboscis. This fits with the observation that OBP56d mRNA is higher in the female than in the male proboscis (Aruçi et al., in preparation). However, such relationships are not always verified. OBP56d expression was also found in the olfactory organs (of the antenna and maxillary palp) and the gustatory sensilla of the anterior wing margin, tarsae, and larval dorsal organs [43]. The higher expression of the OBP56d protein was also found in the female antenna [42], indicating that OBP56d was involved both in taste and olfaction. Moreover, its sex-biased expression suggests that it is involved in the detection of male pheromone(s) in females. The fact that the OBP56d gene is also expressed in non-head tissues, such as the adult hindgut and testis [23], raises the tantalizing hypothesis that this OBP is involved not only in the sensory detection of sex pheromones but also in physiological functions, such as nutrition and reproduction. For instance, two other OBPs are highly related to OBP56d (OBP56f and OBP56g) and have been observed in the male seminal fluid. Both are transferred to the female during copulation to subsequently affect egg-laying behavior [45,46,47]. Another study revealed that the z-7-tricosene (7-T) pheromone elicits a similar electrophysiological signal as bitter substances in the same taste sensilla of the proboscis, suggesting that 7-T tastes bitter to the fly [48]. These two types of molecules induce the dose-dependent inhibition of a male-male homosexual courtship.

Two other *D. melanogaster* OBPs are also involved in sexual behaviors. OBP76a (also named Lush) participates in the olfactory detection of the *cis*-vaccenyl acetate male pheromone (cVA) through a pH-dependent interaction with a sensory neuron membrane protein (SNMP; [49,50]). Lush OBP was initially discovered based on its role in alcohol detection: wild-type flies showed active olfactory avoidance of concentrated alcohol, whereas *lush* mutants did not [51]. Another antenna-expressed OBP (OBP69a), which shows sexually dimorphic expression in *D. melanogaster* flies, also participates in cVA detection. More precisely, the exposure of flies to cVA tended to increase OBP69a levels in females and decrease its levels in males. Increased OBP69a expression led to increased female receptivity and copulation and aggressive behavior in males [52]. These examples suggest that the dimorphic expression of OBP may have a role in fly sexual behavior.

We believe that our proteomic analysis missed several proteins expressed at a minute level in the proboscis. For instance, despite the high number of dissected proboscis, we did not detect OBP19b, which is nevertheless present in this taste organ, given that it is involved in the detection of amino acids by the proboscis taste sensilla [30]. While the evaluation of the level of protein expression based on mRNA expression could be a useful approach in some cases, it remains difficult to carry out, given the possible post-translational modifications and also for technical reasons due to the devices used to detect proteins or mRNA levels. A similar comparison could have been carried out in the antennae given that two studies measured the levels of mRNA and proteins in these appendages [53]. However, this is not possible here, given that no extensive mRNA characterization has yet been carried out on Drosophila proboscis. Other OBPs (OBP56g, OBP19c, OBP83c), which are also present in the proboscis, were not detected in our proteomic screen [28,43]. This indicates that the expression level of these proteins was too low to be detected with our technique. In our study, the lower level of expression was 1.36 × 10^5^ (Cytochrome P450 4d21; Table 1). Protein detection could be limited by masking peptide fragments in nanoLC-MS/MS analyses. This can explain the failure to detect proteins below a detection threshold. A similar bias was observed in the antenna proteomic screening [42]. These authors detected an increased number of OBPs when they used an increased number of antennae in each sample. Another study based on the RNA-seq method was also unable to detect several OBPs, including OBP19d and OBP56d, in the fly antennae [53].

## 5. Conclusions

Our proteomic analysis revealed the presence of many enzymes potentially involved in chemoreception. In particular, we identified putative tastant carriers, including two OBPs. Interestingly, the sexually dimorphic expression observed for OBP56d suggests that it may be involved in the detection and processing of hydrophobic sex-specific pheromone(s) detected by contact. The second (non-sex specific) OBP (OBP19d) is likely involved in food quality detection. Together, these data indicate that the proboscis is a complex organ involved in the detection of general tastant molecules as well as sex pheromones. Further work is needed to characterize the biochemical properties and physiological functions of these OBPs to determine their precise roles in insect chemoreception.

## Figures and Tables

**Table 1:** Proteins that are believed to be involved in the transport of chemicals or that can participate in metabolization and elimination of chemicals present in the proboscis. The mean of the measured abundance of protein is indicated in the right columns.

Accession Number	Protein Identification	Female Mean	Male Mean
sp|Q8SY61|OB56D_DROME	General odorant-binding protein 56d	5.09 × 10^6^	6.41 × 10^5^
sp|P54192|OB19D_DROME	General odorant-binding protein 19d	9.86 × 10^6^	7.10 × 10^6^
sp|Q95RA9|GILT1_DROME	GILT-like protein 1	1.38 × 10^7^	3.45 × 10^6^
sp|Q9V521|PPO2_DROME	Phenoloxidase 2	6.06 × 10^7^	2.79 × 10^7^
sp|Q9V3Z2|SER7_DROME	Serine protease 7	6.70 × 10^5^	4.15 × 10^5^
sp|Q9V3D2|HEM6_DROME	Oxygen-coproporphyrinogen-III oxidase	3.29 × 10^6^	1.26 × 10^6^
sp|Q9W265|HOT_DROME	Hydroxyacid-oxoacid transhydrogenase	1.81 × 10^6^	
sp|O18404|HCD2_DROME	3-hydroxyacyl-CoA dehydrogenase	4.94 × 10^7^	1.90 × 10^7^
sp|Q04499|PROD_DROME	Proline dehydrogenase 1	2.68 × 10^6^	1.71 × 10^6^
sp|Q6AWN0|MTND_DROME	Acireductone dioxygenase	7.14 × 10^5^	1.71 × 10^5^
sp|P36951|HYI_DROME	Putative hydroxypyruvate isomerase	3.57 × 10^6^	1.67 × 10^6^
sp|O77458|TPIS_DROYA	Triosephosphate isomerase	5.76 × 10^7^	2.27 × 10^7^
sp|B3M098|MTNA_DROAN	Methylthioribose-1-phosphate isomerase	1.05 × 10^6^	4.21 × 10^5^
sp|Q7KML2|ACOX1_DROME	Peroxisomal acyl-coenzyme A oxidase 1	4.97 × 10^7^	2.41 × 10^7^
sp|Q9V6U9|MECR_DROME	2-enoyl thioester reductase	2.36 × 10^7^	4.40 × 10^6^
sp|Q01597|G3P_DROHY	Glyceraldehyde-3-phosphate dehydrogenase	2.23 × 10^8^	8.95 × 10^7^
sp|P07487|G3P2_DROME	Glyceraldehyde-3-phosphate dehydrogenase 2	3.49 × 10^8^	1.50 × 10^8^
sp|P07486|G3P1_DROME	Glyceraldehyde-3-phosphate dehydrogenase 1	2.91 × 10^8^	1.30 × 10^8^
sp|O44104|G3P2_DROPS	Glyceraldehyde-3-phosphate dehydrogenase 2	3.70 × 10^8^	1.63 × 10^8^
sp|P13706|GPDA_DROME	Glycerol-3-phosphate dehydrogenase	1.32 × 10^8^	6.49 × 10^7^
sp|O02373|UGDH_DROME	UDP-glucose 6-dehydrogenase	7.76 × 10^5^	3.19 × 10^5^
sp|P54399|PDI_DROME	Protein disulfide-isomerase	9.64 × 10^7^	4.18 × 10^7^
sp|Q9V429|THIO2_DROME	Thioredoxin-2	3.41 × 10^6^	2.07 × 10^6^
sp|Q9VSA3|ACADM_DROME	Medium-chain specific acyl-CoA dehydrogenase	9.71 × 10^7^	4.54 × 10^7^
sp|Q9VWH4|IDH3A_DROME	Isocitrate dehydrogenase	6.06 × 10^7^	5.34 × 10^7^
sp|P32748|PYRD_DROME	Dihydroorotate dehydrogenase	2.65 × 10^5^	1.70 × 10^5^
sp|Q9VXJ0|DHB4_DROME	Peroxisomal multifunctional enzyme type 2	1.86 × 10^7^	1.14 × 10^7^
sp|Q9V3P0|PRDX1_DROME	Peroxiredoxin 1	4.19 × 10^7^	1.84 × 10^7^
sp|P17336|CATA_DROME	Catalase	1.05 × 10^8^	4.20 × 10^7^
sp|Q9VG97|GSTD3_DROME	Glutathione S-transferase D3	6.02 × 10^5^	2.29 × 10^5^
sp|P20432|GSTD1_DROME	Glutathione S-transferase D1	8.75 × 10^7^	4.04 × 10^7^
sp|P41043|GSTS1_DROME	Glutathione S-transferase S1	2.18 × 10^6^	9.93 × 10^5^
sp|Q9VZU4|NDUS3_DROME	NADH dehydrogenase	7.11 × 10^6^	4.10 × 10^6^
sp|Q27597|NCPR_DROME	NADPH--cytochrome P450 reductase	1.31 × 10^6^	1.27 × 10^6^
sp|P07705|NU3M_DROYA	NADH-ubiquinone oxidoreductase chain 3	6.85 × 10^5^	4.46 × 10^5^
sp|P91929|NDUAA_DROME	NADH dehydrogenase	5.74 × 10^6^	2.43 × 10^6^
sp|Q05114|ADH_DROWI	Alcohol dehydrogenase	4.37 × 10^6^	1.82 × 10^6^
sp|B4M8Y0|ADH_DROVI	Alcohol dehydrogenase	7.10 × 10^6^	2.22 × 10^6^
sp|P46415|ADHX_DROME	Alcohol dehydrogenase class-3	5.37 × 10^6^	2.56 × 10^6^
sp|P48584|ADH_DROBO	Alcohol dehydrogenase	2.85 × 10^7^	1.90 × 10^7^
sp|P09369|ADH2_DROMO	Alcohol dehydrogenase 2	5.41 × 10^7^	3.36 × 10^7^
sp|P07161|ADH1_DROMU	Alcohol dehydrogenase 1	5.41 × 10^7^	3.36 × 10^7^
sp|P00334|ADH_DROME	Alcohol dehydrogenase	1.92 × 10^8^	1.30 × 10^8^
sp|P07159|ADH_DROOR	Alcohol dehydrogenase	1.85 × 10^8^	1.25 × 10^8^
sp|P10807|ADH_DROLE	Alcohol dehydrogenase	7.30 × 10^7^	4.76 × 10^7^
sp|Q9W5N0|COA7_DROME	Cytochrome c oxidase	5.51 × 10^5^	2.14 × 10^5^
sp|Q9V9L1|CP6W1_DROME	Cytochrome P450 6w1	7.56 × 10^5^	7.89 × 10^5^
sp|Q9V558|CP4P1_DROME	Cytochrome P450 4p1	3.71 × 10^5^	2.80 × 10^5^
sp|Q9V4N3|CYB5_DROME	Cytochrome b5	1.96 × 10^6^	1.86 × 10^6^
sp|P19967|CYB5R_DROME	Cytochrome b5	1.71 × 10^5^	3.67 × 10^5^
sp|Q9VFP1|CP6D5_DROME	Cytochrome P450 6d5	8.61 × 10^6^	1.08 × 10^7^
sp|Q9VE01|C12A5_DROME	Cytochrome P450 12a5	1.80 × 10^6^	1.85 × 10^6^
sp|P33270|CP6A2_DROME	Cytochrome P450 6a2	3.40 × 10^6^	3.50 × 10^6^
sp|Q9VCW1|CP6D4_DROME	Cytochrome P450 6d4	1.05 × 10^6^	5.61 × 10^5^
sp|Q27606|CP4E2_DROME	Cytochrome P450 4e2	1.90 × 10^5^	2.16 × 10^5^
sp|Q9VFJ0|CA131_DROME	Cytochrome P450 313a1	5.47 × 10^6^	3.35 × 10^7^
sp|Q9VLZ7|C4D21_DROME	Cytochrome P450 4d21	1.36 × 10^5^	3.54 × 10^6^
sp|Q9XY35|QCR9_DROME	Cytochrome b-c1	1.25 × 10^7^	9.11 × 10^6^
sp|Q9VHS2|COX7A_DROME	Cytochrome c oxidase	6.20 × 10^6^	3.43 × 10^6^
sp|P84029|CYC2_DROME	Cytochrome c-2	7.44 × 10^7^	3.46 × 10^7^

**Table 2:** The 19 soluble proteins of Drosophila melanogaster proboscis * Are potentially involved in the transport and elimination of chemical stimuli.

Accession Number	Protein Identification	Protein Mass (kDa)
sp|Q9V3P0|PRDX1_DROME	Peroxiredoxin 1	21.738
sp|P17336|CATA_DROME	Catalase	57.15
sp|Q8SY61|OB56D_DROME	Odorant-binding protein 56d	14.119
sp|P54192|OB19D_DROME	Odorant-binding protein 19d	16.782
sp|Q9VFP1|CP6D5_DROME	**Cytochrome P450 6d5**	57.384
sp|Q9VE01|C12A5_DROME	Cytochrome P450 12a5	61.355
sp|Q9VCW1|CP6D4_DROME	Cytochrome P450 6d4	59.182
sp|Q9VFJ0|CA131_DROME	**Cytochrome P450 313a1**	56.534
sp|Q9V4N3|CYB5_DROME	Cytochrome b5	15.206
sp|Q9W5N0|COA7_DROME	Cytochrome c oxidase	29.64
sp|P84029|CYC2_DROME	Cytochrome c-2 (Cytochrome c-proximal)	11.735
sp|Q9VHS2|COX7A_DROME	Cytochrome c oxidase subunit 7A	9.902
sp|Q9V558|CP4P1_DROME	Cytochrome P450 4p1	59.433
sp|P20432|GSTD1_DROME	Glutathione S-transferase D1	23.866
sp|B4M8Y0|ADH_DROVI	Alcohol dehydrogenase	27.608
sp|P09369|ADH2_DROMO	Alcohol dehydrogenase 2	27.605
sp|P07161|ADH1_DROMU	Alcohol dehydrogenase 1	27.495
sp|P00334|ADH_DROME	Alcohol dehydrogenase	27.761
sp|P46415|ADHX_DROME	Alcohol dehydrogenase 3	40.389

* Underlined entries designate an expression at least two times more important in females than in males; bold font indicates an expression at least two times more important in males than in females.

## Data Availability

All data in this paper were analyzed using the search engine Mascot (version 2.4.0, Matrix Science, London, UK). For the visualization of the results, we used XLSTAT 2022.2.1. The datasets generated and analyzed during this study are available from the corresponding author on request.

## Supplementary Materials

The following supporting information can be downloaded at https://www.mdpi.com/article/10.3390/biology11111687/s1. Table S1: List of identified soluble proteins in proboscis.
